# Characterization of Liver Monocytic Myeloid-Derived Suppressor Cells and Their Role in a Murine Model of Non-Alcoholic Fatty Liver Disease

**DOI:** 10.1371/journal.pone.0149948

**Published:** 2016-02-22

**Authors:** Liying Yao, Masanori Abe, Keitarou Kawasaki, Sheikh Mohammad Fazle Akbar, Bunzo Matsuura, Morikazu Onji, Yoichi Hiasa

**Affiliations:** 1 Department of Gastroenterology and Metabology, Ehime University Graduate School of Medicine, To-on, Ehime, Japan; 2 Department of Medical Sciences, Toshiba General Hospital, Shinagawa, Tokyo, Japan; 3 Department of Internal Medicine, Imabari Saiseikai Medical-Welfare Center, Imabari, Ehime, Japan; National Cancer Institute, UNITED STATES

## Abstract

Myeloid-derived suppressor cells (MDSCs) are potent suppressors of T cell immunity in tumors and inflammatory diseases. They are identified by surface expression of CD11b^+^Gr1^+^ in mice, and CD11b^+^Gr1^+^ cells accumulate in the livers of obese mice. However, many myeloid cells share these CD11b^+^Gr1^+^ markers. Accordingly, the aim of this study was to identify the authentic phenotype of MDSCs and investigate their functions in non-alcoholic fatty liver disease (NAFLD). C57BL/6J mice were divided into 2 diet groups: a normal control group and high-fat group to induce NAFLD. We demonstrated that monocytic CD11b^+^Gr1^dim^ cells could be further divided into 2 populations based on side scatter (SSC) during flow cytometry. We found that SSC^low^CD11b^+^Gr1^dim^ cells accumulated in the livers of NAFLD mice over time, and that these cells were recruited by the chemokine CCL2 and its receptor CCR2 and might expand in the liver via macrophage colony-stimulating factor stimulation. Furthermore, SSC^low^CD11b^+^Gr1^dim^ cells had a strong suppressive ability on T cells; this effect was not observed for SSC^high^CD11b^+^Gr1^dim^ cells, and was dependent on nitric oxide production by inducible nitric oxide synthase. Our findings demonstrate that SSC^low^CD11b^+^Gr1^dim^ cells represent authentic MDSCs in NAFLD livers, and might serve an important negative feedback function in liver inflammation.

## Introduction

Non-alcoholic fatty liver disease (NAFLD) is currently one of the most commonly diagnosed liver diseases worldwide, and includes a wide spectrum of liver pathologies, including simple steatosis, steatohepatitis, liver fibrosis, and cirrhosis [1, 2]. Altered immunomodulation is thought to contribute to the pathogenesis of NAFLD [3]; the T cell-mediated immune response is considered to play a critical role in the associated liver injury [4]. It has been observed that the number of CD4^+^CD25^+^ Treg cells is reduced in obese livers, which leads to impaired suppression of inflammatory responses [5]. M2 macrophages, also categorized as immunosuppressive cells, play a role in limiting liver inflammation and injury in NAFLD [6].

Myeloid-derived suppressor cells (MDSCs) are a heterogeneous population of immature myeloid cells and comprise myeloid precursors of granulocytes, macrophages, and dendritic cells. They accumulate in tumor-bearing hosts, trauma sites, and infections to suppress immune responses via arginase-1, inducible nitric oxide synthase (iNOS), or reactive oxygen species (ROS). In mice, MDSCs were originally defined as CD11b^+^Gr1^+^ cells, whereas in humans, these cells are mainly defined as CD11b^+^CD33^+^CD15^+^HLA-DR^-^ or CD11b^+^CD33^+^CD14^+^HLA-DR^-/low^ cells [7, 8]. However, a specific marker for MDSCs has not yet been described because other myeloid cells share their surface molecules, such as neutrophils, monocytes, and myeloid dendritic cells. Therefore, the most reliable feature that can be used to distinguish MDSCs from other myeloid cells appears to be their suppressive function.

Some studies have indicated that MDSCs play a role in hepatocellular carcinoma (HCC), hepatitis, or liver fibrosis both in patients and mouse models [9–13]. Recently, MDSCs have been found to accumulate in the livers of obese mice to suppress inflammation and maintain liver homeostasis; these MDSCs were identified as CD11b^+^Gr1^+^ [14, 15]. The Gr1 marker is a composite epitope between Ly6C and Ly6G antigens, and MDSCs can be further subdivided into Ly6C^+^ monocytic and Ly6G^+^ granulocytic MDSCs using these 2 antigens [12, 16]. However, other studies have reported that liver CD11b^+^Ly6C^+^ or CD11b^+^Gr1^+^ cells, categorized as macrophages, monocytes, or immature myeloid cells, contribute to liver inflammation [17–19], suggesting that the phenotype of liver MDSCs needs further investigation and specification.

In this study, we successfully elucidated the profile of authentic monocytic MDSCs that accumulated in the livers of NAFLD model mice and assess their function with respect to T cell suppression and their role in the pathogenesis of liver inflammation in NAFLD.

## Materials and Methods

### Mice

Five-week-old male C57BL/6J and C3H/HeN mice were purchased from CLEA Japan (Tokyo, Japan). After 1 week of acclimatization, C57BL/6J mice were divided into 2 groups. The control group was fed a normal diet (13% fat, 26% protein, and 60% carbohydrates; 360 kcal/100 g). The NAFLD group was fed a high-fat diet (60% fat, 20% protein, and 20% carbohydrates; 520 kcal/100 g; D12492; Research Diets, New Brunswick, NJ, USA). The mice were fed these diets for either 3 or 12 months. The NAFLD group fed the high-fat diet for 12 months showed more severe steatosis than those fed the high-fat diet for 3 months. All animals received humane care, and the study protocols were approved by the Institutional Animal Care and Use Committee of Ehime University (No. 05-TI-72-16). Following sacrifice, 10 mg of liver was harvested, submerged in RNA-later (Life Technologies, Carlsbad, CA, USA), and stored at –20°C. Some liver tissue samples were stored at –80°C.

### Cell isolation

Liver non-parenchymal cells and splenocytes were prepared using the procedure described by Chen et al. [20]. T cells were isolated from C57BL/6J mouse splenocytes using the mouse Pan T Cell Isolation Kit II (Miltenyi Biotec, Bergisch Gladbach, Germany). Dendritic cells were isolated from C3H/HeN mouse splenocytes using mouse CD11c Microbeads (Miltenyi Biotec), using an autoMACS Pro Separator (Miltenyi Biotec).

### Flow cytometry and cell sorting

Liver non-parenchymal cell suspensions were pre-incubated with anti-CD16/CD32 (clone 93) to block non-specific FcRγ binding, and then stained with mouse monoclonal antibodies (mAbs) against the following: CD45 (30-F11), Gr1 (RB6-8C5), CD11b (M1/70), Ly6G (1A8), Ly6C (AL-21), CD11c (HL3), CD80 (16-10A1), CD31 (MEC13.3), iNOS (6/iNOS/NOS Type II), and interferon (IFN)-γ (XMG 1.2) (all from BD Biosciences, San Jose, CA, USA), or F4/80 (BM8) (BioLegend, San Diego, CA, USA), CD115 (AFS98) (TONBO Biosciences, San Diego, CA, USA), CD274 (MIH6) (AbD Serotec, Kidlington, UK), and CCR2 (R&D Systems, Minneapolis, MN, USA). For intracellular cytokine staining, cells were lysed using a Fixation and Permeabilization Kit (Invitrogen, Carlsbad, CA, USA) based on the manufacturer's instructions. Flow cytometry was performed on a Becton Dickinson Fluorescence Activated Cell Sorter (FACS) Calibur using CellQuest Software (Becton Dickinson, Franklin Lakes, NJ, USA). Data were analyzed using FlowJo (Tree Star, Ashland, OR, USA). Liver non-parenchymal cells were stained with mAbs specific to CD11b and Gr1, and cell scatter (SSC)^high^CD11b^+^Gr1^dim^ cells and SSC^low^CD11b^+^Gr1^dim^ cells were sorted using the BD FACSAria™ Cell Sorting System (Becton Dickinson). The purity of all sorted cells was greater than 98%.

### MDSC functional assay

To investigate the suppressive function of MDSCs, T cells were stained with 10 μM 5-(and-6)-carboxy-fluorescein diacetate, succinimidyl ester (CFSE) according to the manufacturer`s instructions (Molecular Probes, Carlsbad, CA, USA). CFSE-labeled T cells were cultured with Dynabeads Mouse T-Activator CD3/CD28 (Life Technologies) in the absence or presence of sorted SSC^high^CD11b^+^Gr1^dim^ cells or SSC^low^CD11b^+^Gr1^dim^ cells from the livers of NAFLD mice. After 60 h, T cell proliferation was analyzed by flow cytometry. Division indices were calculated using FlowJo software. To determine the roles of iNOS, ROS, and arginase 1 in T cell proliferation, 0.5 μM _L_-*N*^6^-(1-iminoethyl) lysine dihydrochloride (L-NIL; Sigma-Aldrich, Gillingham, UK), 1000 U/mL catalase (Sigma-Aldrich), or 1 mM *N*-hydroxy-nor-arginine (nor-NOHA; Cayman Chemical, Ann Arbor, MI, USA), was added at the start of the cultures, respectively. In some experiments, T cells were stimulated with phorbol 12-myristate 13-acetate (50 ng/mL; Sigma) and ionomycin (1 μg/mL; Sigma). Allogenic mixed lymphocyte reactions were used to confirm the suppressive ability of MDSCs. T cells from C57BL/6J mice were mixed with dendritic cells from C3H/HeN mice and co-cultured in the absence or presence of sorted SSC^high^CD11b^+^Gr1^dim^ cells or SSC^low^CD11b^+^Gr1^dim^ cells from the livers of NAFLD mice at different ratios. [^3^H]-thymidine (1.0 μCi/mL; Amersham Biosciences, Buckinghamshire, UK) was diluted in sterile RPMI-1640 and added to the cultures for the last 16 h. The stimulation index was calculated using a formula described previously [20]. All culturing was performed in 96-well U-bottomed plates (Corning Inc., New York, NY, USA).

### Nitrite and CCL2 determination

The NO concentration in co-culture supernatants was measured using the Griess Reagent System (Promega, Madison, WI, USA) according to manufacturer’s protocol. For CCL2 determination, protein was extracted from the liver lysate using RIPA buffer supplemented with protease inhibitor cocktail. CCL2 expression was investigated with a RayBio Mouse CCL2 ELISA Kit (RayBiotech, Norcross, GA, USA) according to the manufacturer's protocol.

### *In vitro* MDSC migration assay

Isolated SSC^low^CD11b^+^Gr1^dim^ cells from the livers of NAFLD mice were resuspended at 2 × 10^6^ cells/mL in serum-free RPMI 1640 media. An aliquot of 150 μL of medium containing 0 ng/mL, 10 ng/mL, or 50 ng/mL murine recombinant chemokine ligand 2 (CCL2) (R&D) was added to the feeder tray. Then, an aliquot (100 μL) of the suspension was added to the membrane chamber of the CytoSelect^™^ 96-Well Cell Migration Assay (5 μm, Fluorometric Format; Cell Biolabs, San Diego, CA, USA). The kit was incubated at 37°C for 2 h in a 5% CO_2_ cell culture incubator. Cells that had migrated were stained using CyQUANT GR dye, and fluorescence intensity was measured with a Flex Station 96 ED (Molecular Devices, Sunnyvale, CA, USA). Data are shown in relative fluorescence units.

### Immunohistochemistry

Frozen liver tissues were fixed in neutral-buffered formalin, paraffin-embedded, and cut into 3-μm-thick sections. Sections were dewaxed and rehydrated, and antigens were retrieved by autoclaving for 1 min at 125°C in EDTA buffer (pH 9.0). After washing in phosphate-buffered saline, nonspecific antigens were blocked by incubation of the slides with 1% normal goat serum for 20 min. The sections were then incubated with 1:200 diluted anti-CCL2 antibody (Abcam, Tokyo, Japan) or 1:100 diluted anti-macrophage colony-stimulating factor (M-CSF) antibody (Abcam) at 4°C overnight. Tissue sections were treated with MAX-PO:R (Nichirei, Seattle, WA, USA) for 30 min and then incubated with simple stain DAB solution (Nichirei). Finally, sections were counterstained with hematoxylin, dehydrated, and mounted.

### Hepa1-6 cell culture

The Hepa1-6 cell line, which is derived from a BW7756 tumor from a C57BL/6J mouse, was purchased from DS Pharma Biomedical Japan (Osaka, Japan). To observe the effect of steatosis in hepatocytes, Hepa1-6 cells were exposed to 0.1 mM oleic acid or palmitic acid (Wako Chemical, Osaka, Japan) for 3 h, as described previously [21]. Lipid accumulation was confirmed by Sudan III staining [21]. The concentrations of CCL2 present in culture supernatants were estimated by ELISAs.

### Real-time reverse transcription polymerase chain reaction

RNA was extracted from livers and the Hepa 1–6 cell line using the RNeasy Plus Mini Kit (Qiagen, Hilden, Germany). Reverse transcription reactions were performed using the High-Capacity cDNA Reverse Transcription kit (Applied Biosystems, Foster City, CA, USA), and real-time polymerase chain reaction analysis was performed using SYBR Green I (Roche Diagnostics, Basel, Switzerland) on a LightCycler 480II (Roche Diagnostics). The pairs of sequence-specific primers that were used are listed in S1 Table.

### Induction of bone marrow-derived monocytic MDSCs by M-CSF

Bone marrow cells were obtained by flushing the tibias of C57BL/6J mice, followed by red blood cell lysis. Cells were suspended in complete RPMI 1640 media supplemented with 10% heat-inactivated fetal bovine serum, and cultured with or without recombinant M-CSF (R&D). After 3 days, CD11b^+^Gr1^dim^Ly6C^high^ and CD11b^+^Gr1^dim^Ly6C^low^ cells were sorted to allow testing of their functions in the allogenic mixed lymphocyte reaction assay.

### Statistical analyses

Data were analyzed using JMP 8.0 software (SAS Institute, Cary, NC, USA). Values are presented as means ± SEM. A Student`s *t*-test was employed to compare data between 2 groups. For multiple comparisons, a one-way ANOVA was used. *P < 0*.*05* was considered significant.

## Results

### Expansion of liver SSC^low^CD11b^+^Gr1^dim^ cells by a high-fat diet

**Fig 1:**
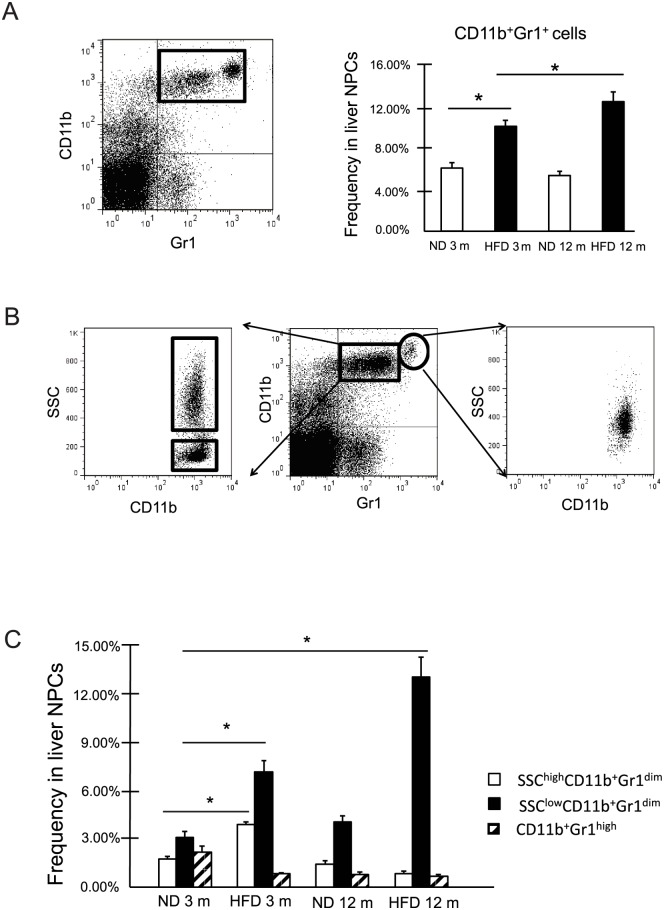
CD11b^+^Gr1^+^ cells in the livers of normal mice and high-fat-diet mice were comprised of 3 subsets. (A) Frequency of CD11b^+^Gr1^+^ cells in mouse liver non-parenchymal cells (NPCs) at 3 months (3 m) or 12 months (12 m) of age (n = 5). *P < 0.*05* (B) Analysis of CD11b^+^Gr1^+^ cells by the side-scatter (SSC) of light during flow cytometry. (C) Frequency of CD11b^+^Gr1^high^, SSC^low^CD11b^+^Gr1^dim^, and SSC^high^ CD11b^+^Gr1^dim^ cells in mouse liver NPCs at 3 m or 12 m of age (n = 5). *P < 0.*05* compared to frequency of each cells at 3 months. ND, normal diet; HFD, high fat diet.

In NAFLD mice, the frequency of liver CD11b^+^Gr1^+^cells was significantly increased compared with that in control mice, as previously reported [14, 15]. We found that the frequency of liver CD11b^+^Gr1^+^cells increased in high-fat diet fed mice over time (Fig 1A). As previously reported [20], these cells could be grouped into 2 subtypes: CD11b^+^Gr1^dim^ and CD11b^+^Gr1^high^. Additional examination by SSC, a measure of cell granularity, revealed that the Gr1^high^ subtype consisted of only 1 population; however, the Gr1^dim^ subtype could be further divided into SSC^high^ and SSC^low^ populations (Fig 1B). The frequency of SSC^high^CD11b^+^Gr1^dim^ cells was increased at 3 months of age in NAFLD mice compared to that in control mice; however, the frequency of these cells was decreased in mice fed with a high-fat diet at 12 months. In NAFLD mice, the frequency of the SSC^low^CD11b^+^Gr1^dim^ cells was significantly higher than that in control mice, and the number of cells increased in the high-fat diet mice over time (Fig 1C).

### Characteristics of CD11b^+^Gr1^+^ cell subsets

**Fig 2:**
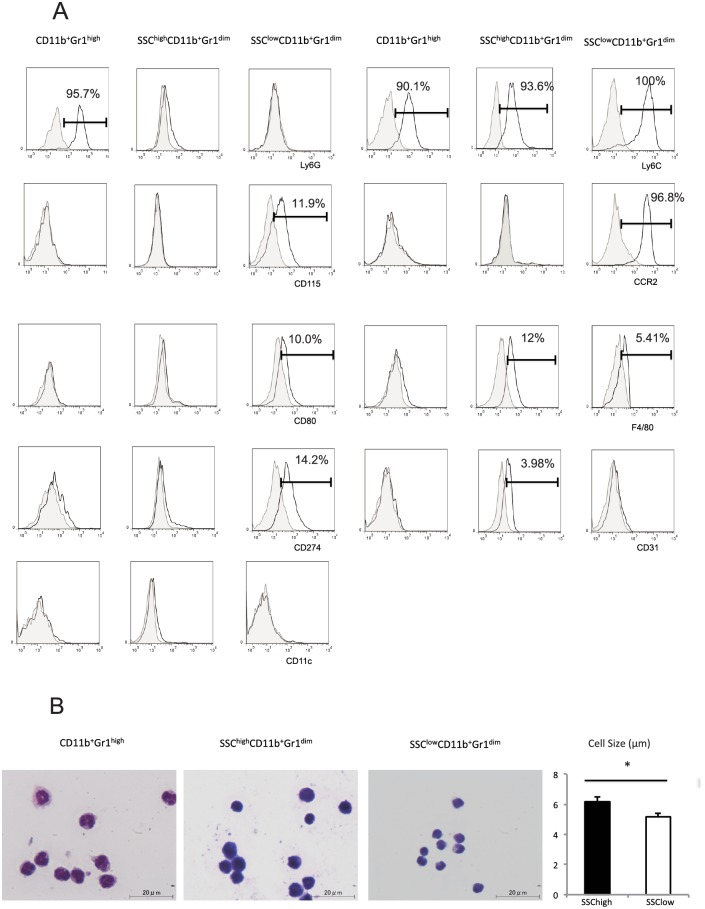
Characterization of liver CD11b^+^Gr1^+^ cell subsets. (A) Representative histograms of the phenotypic profiles of liver CD11b^+^Gr1^high^, SSC^high^CD11b^+^Gr1^dim^, and SSC^low^CD11b^+^Gr1^dim^ cells. Closed histograms represent background control staining; open histograms represent staining by indicated monoclonal antibody (mAb). The percentage of positive cells is indicated. (B) Morphology of purified liver CD11b^+^Gr1^high^, SSC^high^CD11b^+^Gr1^dim^, and SSC^low^CD11b^+^Gr1^dim^ cells by Wright-Giemsa staining (100× magnification). Scale bars, 20 μm. SSC, light side scatter. The sizes of SSC^high^CD11b^+^Gr1^dim^ and SSC^low^CD11b^+^Gr1^dim^ cells are shown in the right panel. *P < 0.*05*

Liver CD11b^+^Gr1^+^ cells were further characterized by examining the expression of cell surface markers and morphology. These cells expressed CD45. SSC^low^CD11b^+^Gr1^dim^ cells expressed Ly6C^high^, whereas SSC^high^CD11b^+^Gr1^dim^ and CD11b^+^Gr1^high^ cells expressed Ly6C^low^. Ly6G was detected only on CD11b^+^Gr1^high^ cells. CCR2, CD115, CD274, and CD80 were detected only on SSC^low^CD11b^+^Gr1^dim^ cells, and CD31 was detected only on SSC^high^CD11b^+^Gr1^dim^ cells. F4/80 was detected on both SSC^high^ and SSC^low^ CD11b^+^Gr1^dim^ cells; however, the expression level in the SSC^high^ population was higher than that in the SSC^low^ population. None of subsets expressed CD11c (Fig 2A). Wright-Giemsa staining demonstrated that CD11b^+^Gr1^high^ cells had lobular-shaped nuclei, typical of granulocytes, whereas SSC^high^CD11b^+^Gr1^dim^ and SSC^low^CD11b^+^Gr1^dim^ cells had ovoid nuclei, typical of monocytes/macrophages. Additionally, the SSC^high^ population was larger in size than the SSC^low^ population (SSC^high^ population: 6.81 ± 0.31 μm; SSC^low^ population: 5.17 ± 0.25 μm), as shown in Fig 2B (n = 5; *p < 0*.*05*).

### Immunosuppressive function of SSC^low^CD11b^+^Gr1^dim^ cells

**Fig 3:**
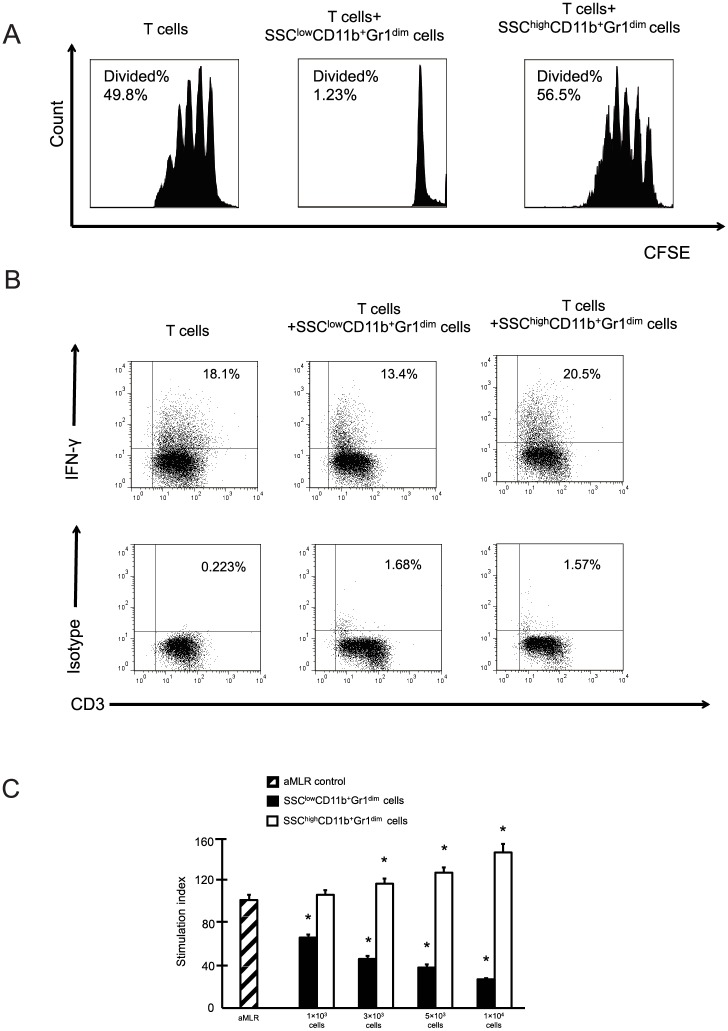
SSC^low^CD11b^+^Gr1^dim^ cells in the livers suppress T cell responses. (A) Proliferation of CFSE-labeled T cells cultured in the presence of Dynabeads mouse T-Activator CD3/CD28 with or without liver SSC^high^ or SSC^low^ CD11b^+^Gr1^dim^ cells. (B) Representative image of intracellular interferon-γ staining for T cells cultured with or without liver SSC^high^ or SSC^low^ CD11b^+^Gr1^dim^ cells. (C) T cells and allogenic dendritic cells were co-cultured. Liver SSC^high^ and SSC^low^CD11b^+^Gr1^dim^ cells were added to the cultures. The data obtained from 3 separate experiments are shown. *P < 0.*05* compared to the levels of T cell proliferation in allogenic mixed lymphocyte reactions (aMLRs) without CD11b^+^Gr1^dim^ cells.

We found that liver SSC^low^CD11b^+^Gr1^dim^ cells suppressed T cell proliferation; however, SSC^high^CD11b^+^Gr1^dim^ cells did not affect T cell proliferation (Fig 3A). In addition, the frequency of IFN-γ-producing T cells decreased after co-culturing with SSC^low^CD11b^+^Gr1^dim^ cells (Fig 3B). Based on allogenic mixed lymphocyte reaction assays, liver SSC^low^CD11b^+^Gr1^dim^ cells suppressed allogeneic T cell proliferation in a dose-dependent manner. The suppressive activity was remarkably effective, even at a ratio of 1:200. Similar to the results shown in Fig 3A, SSC^high^CD11b^+^Gr1^dim^ cells exhibited no suppressive ability in the allogenic mixed lymphocyte reactions (Fig 3C).

### SSC^low^CD11b^+^Gr1^dim^ MDSCs inhibit T cell proliferation via a NO-dependent mechanism

**Fig 4:**
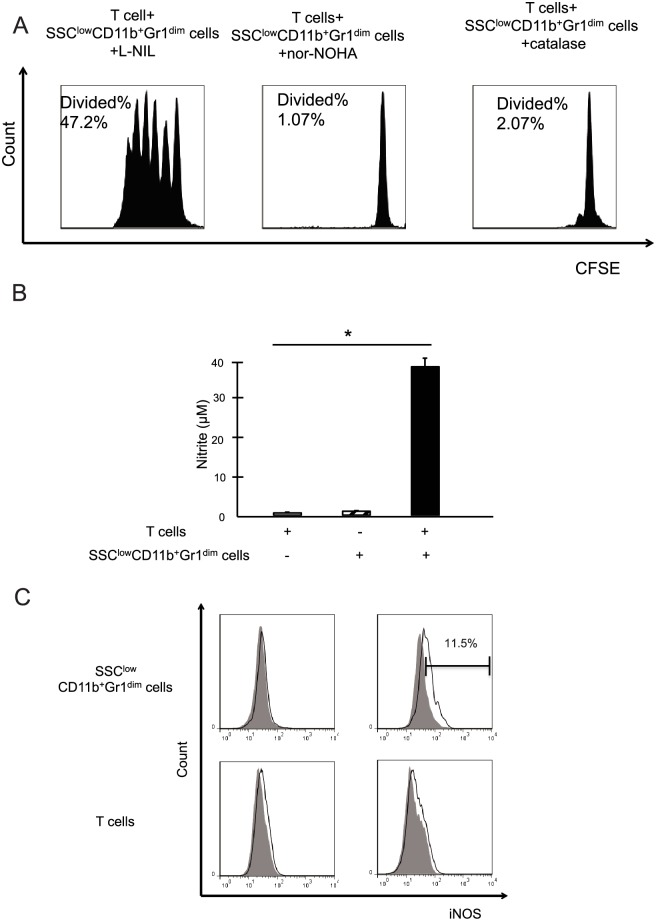
The suppressive function of liver SSC^low^CD11b^+^Gr1^dim^ cells is dependent on a NO mechanism. (A) Proliferation of carboxy-fluorescein diacetate, succinimidyl ester (CFSE)-labeled T cells cultured in the presence of Dynabeads mouse T-Activator CD3/CD28 along with purified liver SSC^low^CD11b^+^Gr1^dim^ cells. Different enzyme inhibitors (L-NIL, nor-NOHA, or catalase) were added at the start of each respective culture. (B) Nitrite levels were investigated in the culture supernatants after 60 h of co-culture (n = 3). (C) The intracellular iNOS expression was determined by flow cytometry. The percentage of positive cells is indicated. The data obtained from 3 separate experiments are shown. *P < 0.*05* compared to T cells alone.

iNOS, arginase 1, or ROS inhibitor was added to the co-culture. Neither nor-NOHA (an arginase 1 inhibitor) nor catalase (a ROS inhibitor) affected T cell proliferation. However, the addition of L-NIL, an iNOS inhibitor, restored T cell proliferation (Fig 4A). The level of nitrite in the supernatants of T cells or liver SSC^low^CD11b^+^Gr1^dim^ MDSCs alone was below the threshold of detection. However, after T cells and liver SSC^low^CD11b^+^Gr1^dim^ MDSCs were co-cultured, nitrite was produced in the supernatants at detectable levels (Fig 4B). Liver SSC^low^CD11b^+^Gr1^dim^ MDSCs cultured alone did not express iNOS, whereas after they were co-cultured with T cells, iNOS expression was detected. The T cells had no independent ability to express iNOS (Fig 4C). These results indicated that the role of liver SSC^low^CD11b^+^Gr1^dim^ cells is distinct from that of their cognate SSC^high^CD11b^+^Gr1^dim^ cells, representing an authentic phenotype of monocytic MDSCs in the liver.

### SSC^low^CD11b^+^Gr1^dim^ MDSCs might be recruited to the NAFLD liver via the CCL2-CCR2 pathway

**Fig 5:**
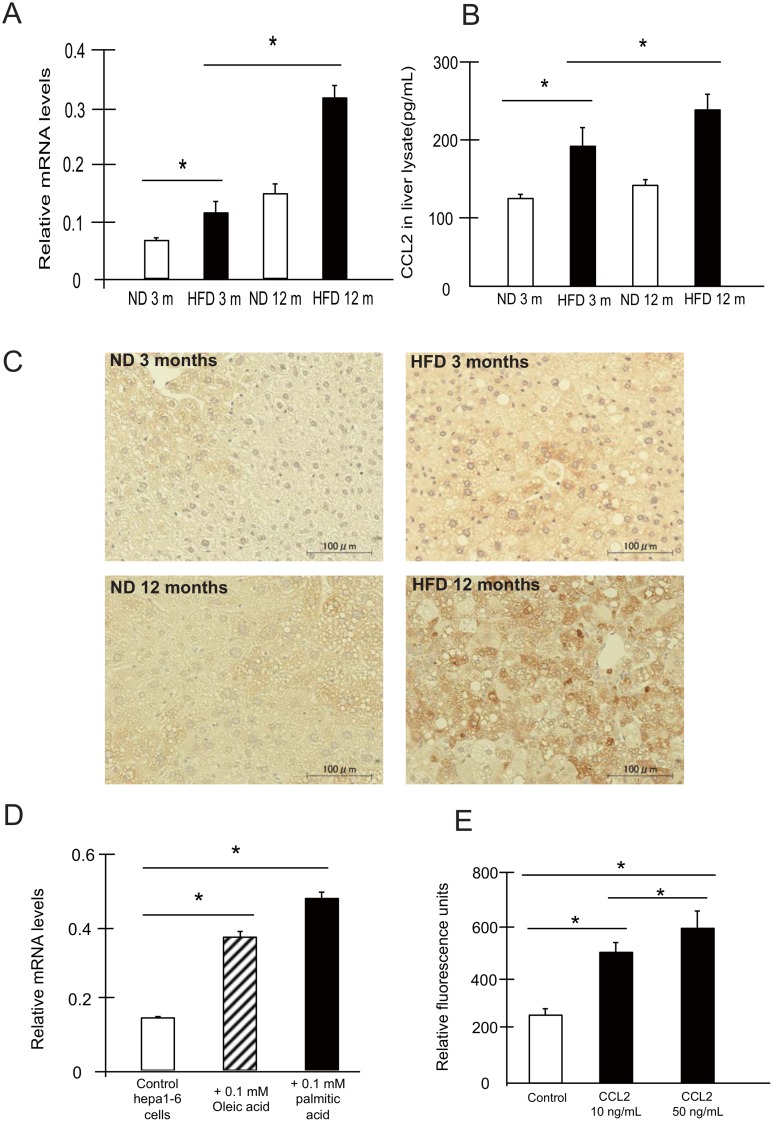
The CCL2/CCR2 pathway mediates the migration of SSC^low^CD11b^+^Gr1^dim^ cells to the NAFLD liver. (A) *CCL2* expression in the livers of ND and HFD-fed mice was investigated by real-time RT-PCR (n = 5). (B, C) Protein expression of CCL2 in the livers was investigated by ELISA (B) (n = 5) and immunohistochemistry (C). Scale bars, 100 μm. (D) *CCL2* gene expression in Hepa1-6 cells treated with oleic acid or palmitic acid analyzed by real-time RT-PCR (n = 5). (E) Migration assays revealed that SSC^low^CD11b^+^Gr1^dim^ cells migrated in response to CCL2 *in vitro* (n = 5). *P < 0.*05*

Chemokines recruit immune cells to inflamed sites. As shown in Fig 2, SSC^low^CD11b^+^Gr1^dim^ MDSCs expressed the receptor for the chemokine CCL2, CCR2. We found that the *CCL2* mRNA expression level was increased in the livers of NAFLD mice (Fig 5A), as was CCL2 protein expression (Fig 5B and 5C). Although hepatocytes treated with normal diet for 12 months showed some changes in fat contents, this finding may have been related to age-induced triglyceride accumulation [22]. As some studies have generated hepatocyte steatosis models by culturing cells with fatty acids (oleic and palmitic acid) *in vitro* [23, 24], we confirmed these results using Hepa1-6 cells supplemented with 0.1 mM oleic acid or palmitic acid. *CCL2* mRNA expression increased with lipid accumulation *in vitro* (Fig 5D). In addition, CCL2 protein secretion from Hepa 1–6 cells (558.2 ± 22.8 pg/ml) increased compared with that from cells treated with oleic acid (605.2 ± 13.1 pg/ml) or palmitic acid (590.4 ± 8.7 pg/ml) (n = 5; *p < 0*.*05*). These data suggest that steatosis induces increased CCL2 expression in hepatocytes. Finally, we evaluated the migratory capacity of SSC^low^CD11b^+^Gr1^dim^ MDSCs, and found that these cells migrated in response to CCL2 in a dose-dependent manner (Fig 5F).

### Induction of SSC^low^CD11b^+^Gr1^dim^ MDSCs in the NAFLD liver might be associated with M-CSF upregulation

**Fig 6:**
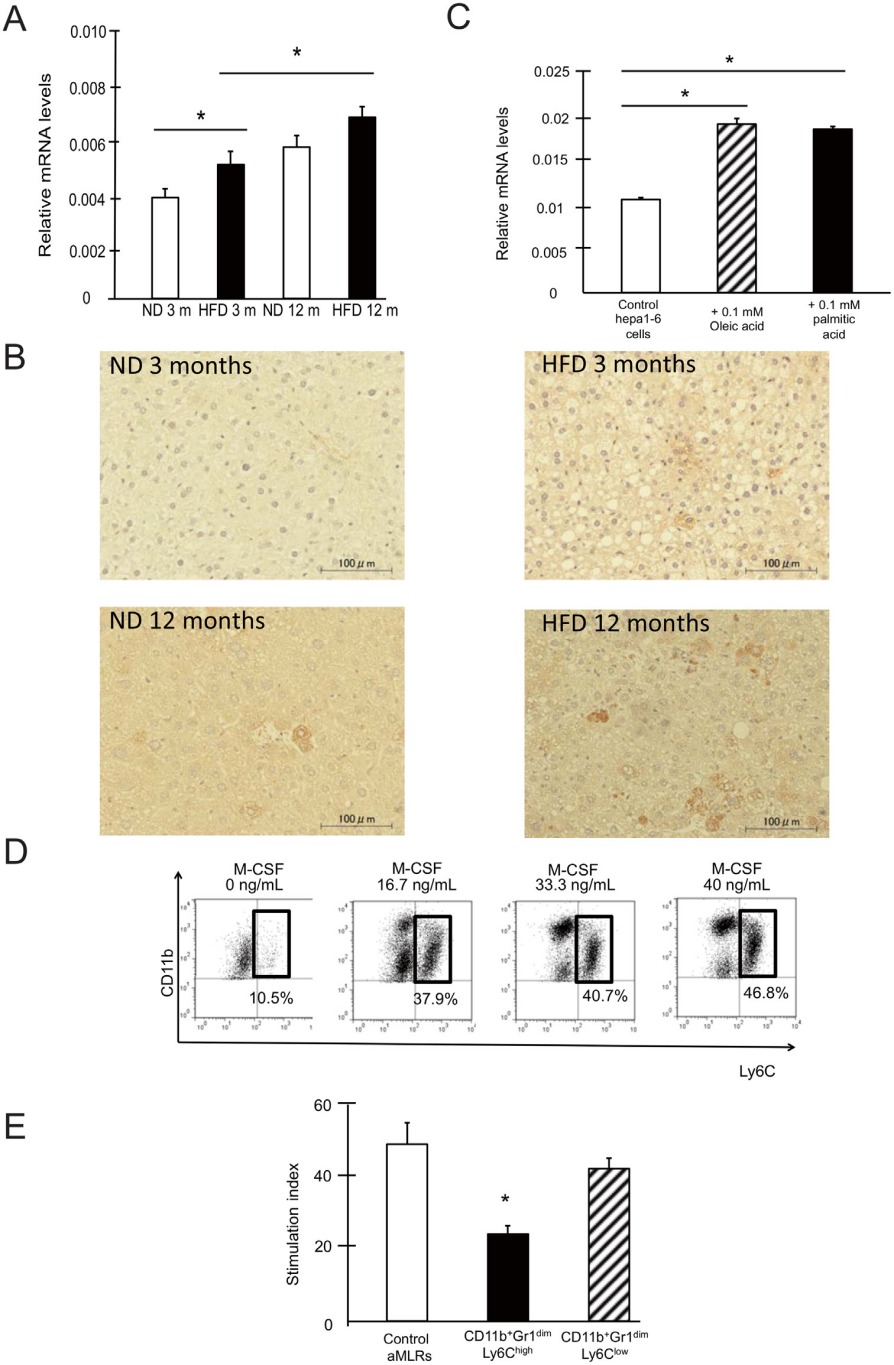
M-CSF is associated with SSC^low^CD11b^+^Gr1^dim^ cell expansion in the liver. (A) Liver M-CSF mRNA was investigated by real-time RT-PCR (n = 5). (B) Liver M-CSF protein was investigated by immunohistochemistry (n = 5). Scale bars, 100 μm. (C) *M-CSF* mRNA in Hepa1-6 cells treated with fatty acid was analyzed by real-time RT-PCR (n = 5). (D) Bone marrow cells were cultured with M-CSF. The frequency of CD11b^+^Gr1^dim^Ly6C^high^ cells among the CD11b^+^Gr1^dim^ cells is shown. (E) The Ly6C^high^ and Ly6C^low^ populations (S1 Fig) were sorted and added to allogenic MLRs. *P < 0.*05*

SSC^low^CD11b^+^Gr1^dim^ MDSCs expressed the M-CSF receptor CD115. M-CSF has previously been shown to play an important role in the development and induction of MDSCs [7]. We found that the mRNA expression of *M-CSF* was higher in the livers of NAFLD mice than control mice (Fig 6A). The protein expression of M-CSF was also increased in hepatocytes, as well as in non-parenchymal cells of NAFLD mouse livers (Fig 6B). The increased expression of M-CSF was confirmed using Hepa1-6 cells cultured with fatty acid (oleic or palmitic acid) (Fig 6C), indicating that steatotic hepatocytes produce M-CSF. To confirm the role of M-CSF in the induction of MDSCs, we cultured bone marrow cells with or without recombinant M-CSF for 3 days. CD11b^+^Gr1^dim^ cells in the bone marrow were divided into 2 subtypes, which showed low (Ly6C^low^) or high (Ly6C^high^) Ly6 expression. CD11b^+^Gr1^dim^Ly6C^high^ cells in the bone marrow are phenotypically similar to SSC^low^CD11b^+^Gr1^dim^ MDSCs in the liver (S1 Fig). These cells showed an increased prevalence in cultures grown in the presence of M-CSF in a dose-dependent manner (Fig 6D). In addition, CD11b^+^Gr1^dim^Ly6C^high^ these cells, but not other CD11b^+^Gr1^dim^ cells in the bone marrow, exhibited immunosuppressive ability (Fig 6E).

## Discussion

The liver is a primary site for MDSC induction and accumulation. Recently, several studies have linked these cells to the pathogenesis of HCC, hepatitis, or liver fibrosis [9,10,12]. In this study, we found that liver CD11b^+^Gr1^dim^ cells, which are monocytic MDSCs in general, could be divided into 2 phenotypes, SSC^high^ and SSC^low^ populations. SSC^low^CD11b^+^Gr1^dim^ cells accumulated in the livers of NAFLD mice (Fig 1). These enlarged SSC^low^ populations had a strong suppressive effect against T cells (Fig 3) and expressed Ly6C^high^, CCR2, CD115, CD80, and CD274 (Fig 2A), which are characteristic of MDSCs in tumors [16, 25]. Morphologic assessment showed that these were monocyte-derived cells (Fig 2B). In contrast, the SSC^high^ populations expressed Ly6C^low^, F4/80, and CD31 (Fig 2A). Although CD31 was found to be a marker of MDSCs in some previous studies [20, 26], the SSC^high^ populations activated T cell proliferation (Fig 3). CD11b^+^Gr1^high^ cells expressed Ly6G^high^ and Ly6C^low^ antigens and had a granulocytic morphology (Fig 2). Because this population was present at a very low level in the livers of NAFLD mice, we did not investigate their function in T cell proliferation. However, some studies have shown that these granulocytic CD11b^+^Gr1^high^ cells from mouse livers also exert a suppressive effect on T cells, but that this suppressive activity is weaker than that of CD11b^+^Gr1^dim^ cells [20, 27].

Obesity is a chronic low-grade inflammatory condition. Lymphocytes are often detected in the lobular infiltrates of obese livers [28], and it is believed that these cells contribute to the progression of NAFLD, which is associated with an increased production of cytokines and exacerbated liver parenchymal injury and fibrosis [4]. The depletion of liver MDSCs has been shown to enhance fibrosis markers [12], suggesting a protective role for MDSCs in liver fibrosis. Other studies have demonstrated that MDSCs exhibit protective and immunosuppressive properties during host infection [11, 29]. Concordantly, the results of this study revealed that liver SSC^low^CD11b^+^Gr1^dim^ MDSCs have a strong suppressive effect on T cells (Fig 3). The accumulation of SSC^low^CD11b^+^Gr1^dim^ MDSCs in livers of NAFLD might allow them to function as critical “homeostatic” regulators to counteract proinflammatory cells. Depletion of SSC^low^ MDSCs may provide important information regarding their contribution to NAFLD. However, antibodies or pharmacological inhibitors to specifically target these cells have not been established, as of yet. Future studies regarding this point need to be conducted.

At the mechanistic level, the suppressive activity of MDSCs has been associated with L-arginine metabolism. L-Arginine is a substrate for iNOS, which is highly expressed in MDSCs [7]. NO production via this pathway is a powerful modulator of inflammation and has been reported to preferentially inhibit T cell immune responses [30, 31]. NO suppresses T cell function by blocking the activation of several important signaling molecules in T cells [32]. NO has also been shown to suppress MHC class II expression and promote T cell apoptosis [33, 34]. Our study demonstrated that liver SSC^low^CD11b^+^Gr1^dim^ MDSC inhibition of T cell proliferation is dependent on NO production by iNOS (Fig 4), consistent with these studies. However, further research is necessary to clarify the mechanisms of iNOS induction in MDSCs that occurs after co-culture with T cells.

The results of several studies provide a link between chemokines and MDSC accumulation in the liver in HCC [35, 36]. The CCL2/CCR2 chemokine axis plays a pivotal role in the migration of MDSCs in cancer, and impairment of CCL2/CCR2 signaling inhibits tumor growth [37–39]. In this study, we found that the expression of CCL2 was up-regulated in the livers of NAFLD mice, and that CCL2 could stimulate the migration of SSC^low^CD11b^+^Gr1^dim^ MDSCs *in vitro* (Fig 5). Thus, the CCL2-CCR2 pathway might contribute to SSC^low^CD11b^+^Gr1^dim^ MDSC accumulation in the steatotic liver.

M-CSF regulates the proliferation, differentiation, chemotaxis, and survival of mononuclear phagocytic cells through its action on CD115 [40]. Recent studies have shown that M-CSF expression is correlated with the expansion of MDSCs [7], and that blocking CD115 inhibits the immunosuppressive tumor milieu and facilitates immune responses, resulting in improved antitumor T-cell function [41]. In this study, we found that M-CSF expression was higher in the livers of NAFLD mice than control mice. In addition, M-CSF induced the expansion of monocytic MDSCs *in vitro* (Fig 6). These results suggest that the increased level of M-CSF in the steatotic liver might contribute to the increase in the frequency of SSC^low^CD11b^+^Gr1^dim^ MDSCs.

Previous data have shown that MDSCs accumulate in mouse models of HCC and may play critical roles in the immune escape of tumor cells [7]. Obesity and NAFLD are recognized as major risk factors of HCC [42]. Although further studies are necessary, it appears likely that the increased frequency of MDSCs in NAFLD contributes to HCC pathogenesis. Recently, an increased frequency of MDSCs in NAFLD patients was reported [43]. Additional clinical research regarding the role of MDSCs in HCC and NAFLD should be pursued.

In conclusion, we identified SSC^low^CD11b^+^Gr1^dim^ as the authentic phenotype of liver monocytic MDSCs and showed that these exhibit a strong suppressive effect on T cells. In addition, these cells inhibited T cells via NO production by iNOS. Our results suggest that the accumulation of MDSCs in the liver might regulate the immune environment of NAFLD.

## Supporting Information

S1 Fig Subtypes of CD11b^+^Gr1^dim^ cells in the bone marrow and liver NPCs. (A) Subtypes of CD11b^+^Gr1^dim^ cells in the bone marrow after culturing in the presence of M-CSF. (B) Subtypes of CD11b^+^Gr1^dim^ cells in liver NPCs.
(EPS)

S1 Table Primer sequences for real-time reverse transcription-polymerase chain reaction amplification. (DOCX)
