# Supplementary material for: Characterization of Liver Monocytic Myeloid-Derived Suppressor Cells and Their Role in a Murine Model of Non-Alcoholic Fatty Liver Disease
Source: PLoS One. 2016 Feb 22;11(2):e0149948. doi: 10.1371/journal.pone.0149948 (PMC4762771; doi:10.1371/journal.pone.0149948)
Supplement: S1 Table — (DOCX) [file pone.0149948.s002.docx]

| **Gene** | **Accession No.** | **Primer sequence** | **Length** | **Position** | **Annealing temperature (°C)** |
| --- | --- | --- | --- | --- | --- |
|  |  | 5'-forward-3' |  |  |  |
|  |  | 5'-reverse-3' |  |  |  |
| *GAPDH* | NM_008084 | TGCACCACCAACTGCTTA | 18 | 498–515 | 63 |
|  |  | GGATGCAGGGATGATGTT | 18 | 657–674 |  |
| *MCP-1* | NM_011333 | CATCCACGTGTTGGCTCA | 18 | 139–156 | 70 |
|  |  | GATCATCTTGCTGGTGAATGAGT | 23 | 192–214 |  |
| *M-CSF* | NM_007778 | CAGCTGCTTCACCAAGGACT | 20 | 766–785 | 65 |
|  |  | TCATGGAAAGTTCGGACACA | 20 | 806–825 |  |

**S1 Table**

Primer sequences for real-time reverse transcription-polymerase chain reaction amplification.
